# Identification of candidate genes and molecular mechanisms related to asthma progression using bioinformatics

**DOI:** 10.1007/s11325-024-03122-0

**Published:** 2024-08-01

**Authors:** Songbing Zou, Fangchan Meng, Guien Xu, Rongchang Yu, Chaomian Yang, Qiu Wei, Yanlong Xue

**Affiliations:** Department of Pulmonary and Critical Care Medicine, The First People’s Hospital of Nanning, Guangxi, China

**Keywords:** Moderate asthma, Severe asthma, T cell immune, Candidate genes, WGCNA

## Abstract

**Background:**

Asthma is a heterogeneous disorder. This study aimed to identify changes in gene expression and molecular mechanisms associated with moderate to severe asthma.

**Methods:**

Differentially expressed genes (DEGs) were analyzed in GSE69683 dataset among moderate asthma and its controls as well as between severe asthma and moderate asthma. Key module genes were identified via co-expression analysis, and the molecular mechanism of the module genes was explored through enrichment analysis and gene set enrichment analysis (GSEA). GSE89809 was used to verify the characteristic genes related to moderate and severe asthma.

**Results:**

Accordingly, 2540 DEGs were present between moderate asthma and the control group, while 6781 DEGs existed between severe asthma and moderate asthma. These genes were identified into 14 co-expression modules. Module 7 had the highest positive correlation with severe asthma and was recognized to be a key module by STEM. Enrichment analysis demonstrated that the module genes were mainly involved in oxidative stress-related signaling pathways. The expression of HSPA1A, PIK3CG and PIK3R6 was associated with moderate asthma, while MAPK13 and MMP9 were associated with severe asthma. The AUC values were verified by GSE89809. Additionally, 322 drugs were predicted to target five genes.

**Conclusion:**

These results identified characteristic genes related to moderate and severe asthma and their corresponding molecular mechanisms, providing a basis for future research.

**Supplementary Information:**

The online version contains supplementary material available at 10.1007/s11325-024-03122-0.

## Introduction

Asthma is a common respiratory disease characterized by wheezing, nocturnal cough, shortness of breath, chest tightness, and limited expiratory capacity [1]. It is estimated that 300 million people suffer from asthma globally [2]. Asthma, a non-communicable disease, can lead to a poor quality of life, poor physical function, and reduced emotional health. The severity of asthma varies greatly between individuals and within individuals over time [3]. The severity of asthma varies from mild to severe, and more severe asthma is associated with a severe incidence and mortality rate [4]. Many new treatments for severe asthma have been investigated and trialed, however, the molecular characteristics of the disease are not yet known.

Asthma is characterized by allergy, airway hyperresponsiveness, inflammation, airway remodeling, and increased immune cells in the airway [5]. Many signaling pathways are involved in the development of asthma, including ER stress, GATA 3, and Janus kinase/signal transducer and transcription (JAK-STAT) pathways [6, 7]. Studies on different degrees of asthma have consistently showed that about 50% of each group showed type 2 inflammatory signals [8, 9]. However, in asthma patients with lesser severity, the type 2 inflammatory process was related to early-onset allergic disease, whereas in severe asthma, the incidence of allergic disease was low with its relationship to allergic disease was unclear [10]. Interestingly, neutrophils are also associated with the severity of asthma [11]. The mechanistic difference between mild asthma and severe asthma can partly be attributed to the apparent infiltration of T cells [12]. In many patients with mild to moderate asthma, airway remodeling is reversible, but in patients with severe asthma, remodeling is chronic [13]. The concept of inflammatory heterogeneity is essential for the emergence of treatments that appear to be effective in the target population for severe asthma [14].

Significant progress has been made in understanding asthma. However, challenges remain in its diagnosis, evaluation, treatment selection, confirmation of compliance and evaluation of contributing diseases. In order to improve the prognosis of patients suffering from varying degrees of asthma, a clear method must be established so as to diagnose patients with asthma. Biomarkers may serve as a solution in determining patient characteristics to predict the prognosis and treatment response. In this study, biomarkers and potential therapeutic targets were identified through gene expression changes in patients with moderate and severe asthma in order to identify the molecular mechanisms related to differences in severity.

## Materials and methods

### Data sources and differentially expressed genes (DEGs)

The GSE69683 and GSE89809 datasets were collected from the gene expression omnibus (GEO) database of NCBI. GSE69683 included 78 healthy controls, 77 moderate asthmatics and 334 severe asthmatics. GSE89809 included 15 healthy controls, 14 moderate asthmatics and 11 severe asthmatics. Participants were categorized based on asthma severity according to the Global Initiative for Asthma (GINA) guidelines, considering factors such as symptom frequency, FEV1% predicted, and exacerbation history. The R package ‘Affy’ was used to preprocess and standardize the datasets. The differentially expressed genes between moderate asthma and the control, as well as severe asthma and moderate asthma, were calculated by R-Pack ‘limma’. The threshold value of *p* < 0.05 was set.

### Weighted gene co-expression network analysis (WGCNA)

Weighted Gene Co-expression Network Analysis (WGCNA) was performed using the R package ‘WGCNA’. The analysis began with the selection of a soft-thresholding power β, aimed at achieving a scale-free topology index of 0.9, to construct a weighted network. The automatic network construction function, along with a minimum module size of 30 genes and a merge cut height of 0.25, was used to identify modules of highly correlated genes. Modules were then related to clinical traits (moderate and severe asthma) using gene significance (GS) and module membership (MM) measures. The module showing the highest correlation with the trait of interest was further analyzed. Hub genes within each module were identified based on their Gene Significance (GS) and Module Membership (MM) scores, with threshold values set to identify genes most central to module connectivity and most correlated with the clinical trait. Hub gene was the abbreviation for “highly linked gene”.

### Enrichment analysis and gene set enrichment analysis (GSEA)

The genes in the module were extracted for further functional enrichment analysis. The R package ‘clusterProfiler’ was used to perform Gene Ontology (GO) and Kyoto Encyclopedia of Genes and Genomes (KEGG) pathway enrichment analyses. The threshold value of *p* < 0.05 was set.

### Receiver operating characteristic (ROC) curve

The ROC analysis was conducted using the R package ‘pROC’. This method assessed the diagnostic ability of identified biomarkers (genes) to distinguish between moderate and severe asthma cases. For each gene, an AUC value was calculated, where a value closer to 1 indicated a better diagnostic performance.

### Drug prediction

To identify potential drugs targeting the hub genes associated with asthma severity, a query was made to the Drug-Gene Interaction Database (DGIdb, https://www.dgidb.org/). This database was searched using the names of the hub genes to find approved or experimental drugs with known interactions. Filters were applied to exclude drugs without direct gene interaction evidence or those not related to respiratory conditions. The results were further categorized based on the mechanism of action and therapeutic class, providing a focused list of potential therapeutic agents for further investigation.

## Results

### Construction of a co-expression module with DEGs

**Fig. 1:**
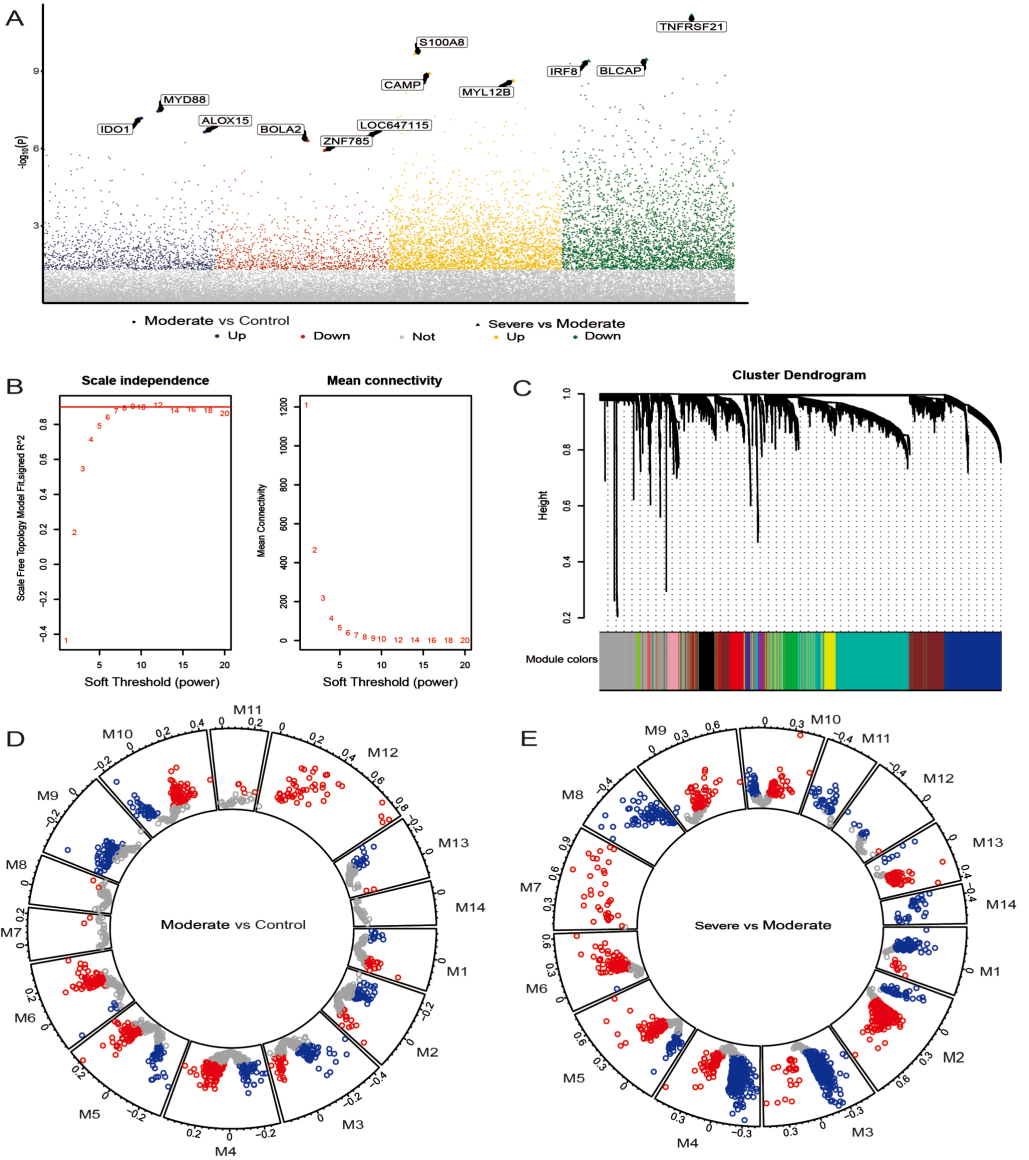
Co-expression network of differentially expressed genes. (**A**) The DEGs between moderate asthma and the control as well as between severe asthma and moderate asthma. (**B**) Soft-thresholding powers of WGCNA. (**C**). Gene clustering tree, where each color represents a module. (**D**) The distribution of DEGs between moderate asthma and the control in modules. E. The distribution of DEGs between severe asthma and moderate asthma in modules

In order to screen different levels of asthma related genes, the dataset containing the gene expression profile of moderate and severe asthma patients was downloaded from the GEO database. Here, 2540 DEGs were present between moderate asthma patients and the healthy controls. Moreover, 6781 DEGs existed among patients with severe asthma and patients with moderate asthma (Fig. 1A). The co-expression network was constructed by combining the two groups of DEGs. The soft threshold power β was set to 8 according to approximate scale free topology in the subsequence analysis (Fig. 1B). A total of 14 modules with similar gene expression characteristics were identified, and their hub genes were then identified (Fig. 1C; Table 1). The distribution of DEGs in the module was different between the two groups of DEGs. Module 9 only contained moderately related downregulated DEGs, while modules 7, 8, 11 and 12 only contained upregulated DEGs (Fig. 1D). In module 7, only upregulated DEGs between severe and moderate were included, while in modules 8, 11 and 14, only downregulated DEGs (Fig. 1E) were included. In addition, the heatmap showed DEGs in the module of patients with moderate and severe asthma (Figure S1). The expression trends in regard to severe and moderate DEGs among the modules were observed to be significantly different.

**Table 1:** Hub genes of module

Colour	HubGenes	Module
black	WDFY3	m6
blue	POM121L9P	m2
brown	PTCD3	m3
cyan	IGLJ3	m14
green	FBXO33	m10
greenyellow	SLC29A1	m12
magenta	FCRLA	m8
pink	GMPR	m9
purple	RPS7	m13
red	FARSA	m1
salmon	CEACAM6	m7
tan	XAF1	m11
turquoise	HNRNPR	m4
yellow	XPO6	m5

### Module correlation and key module identification

**Fig. 2:**
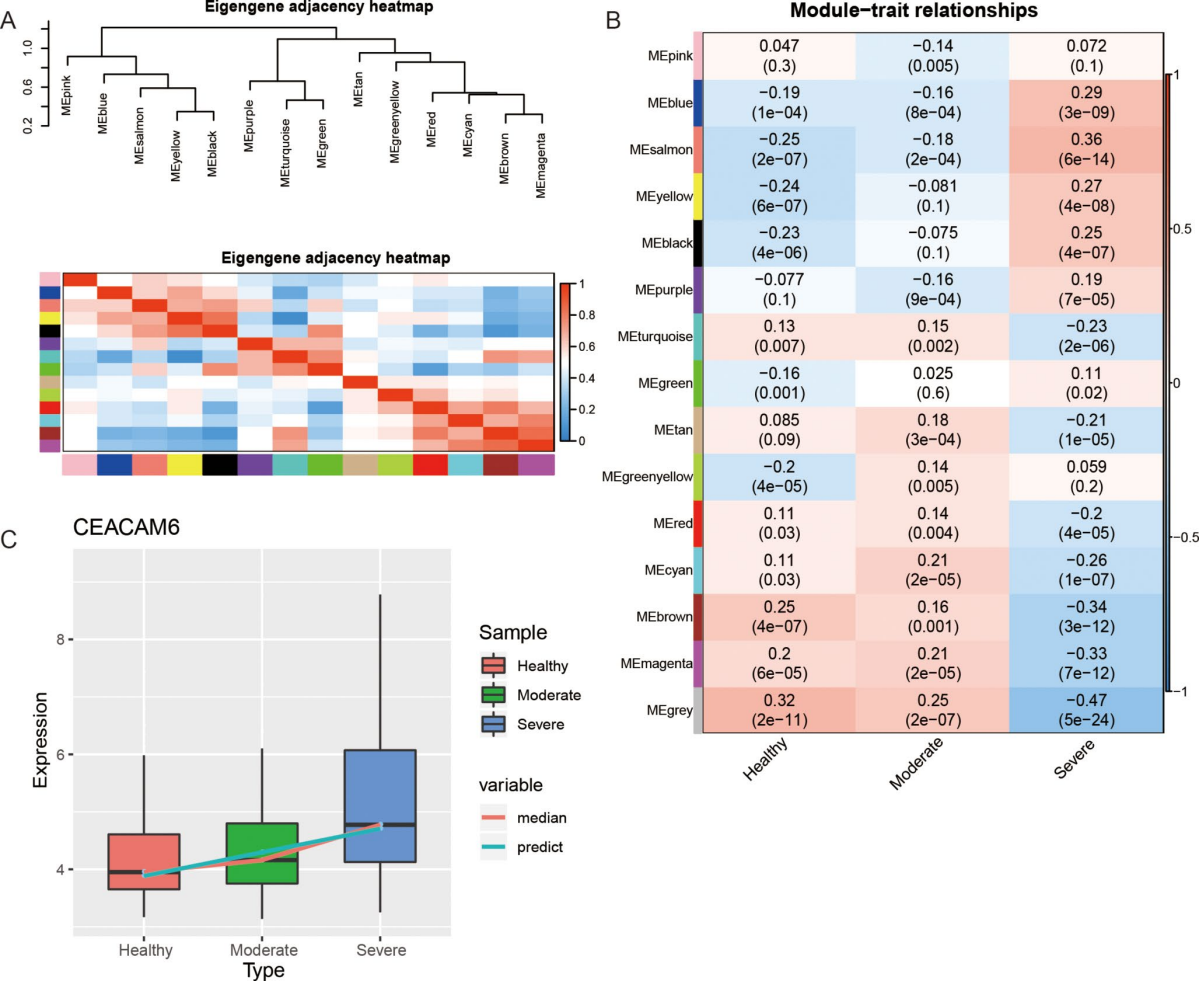
Module correlation and key module. (**A**) The cluster tree and heatmap of the co-expression modules. (**B**). Thermogram of the correlation between modules and clinical phenotypes. Red represents a positive correlation and blue represents a negative correlation. (**C**). Expression of hub gene CEACAM6 of key module 7 among the three groups

In order to further explore the relationship between modules, the correlation between modules was calculated (Fig. 2A). The cluster tree divided the modules into two categories, where module genes in the same category possessed a certain similarity in expression trends. The key module was identified by associating the module with the clinical phenotype (Fig. 2B). The genes in the grey module could not be divided into any modules and was consequently removed. The results demonstrated that MEbrown (module 3) had the strongest positive correlation with the control group as well as the strongest negative correlation with severe asthma. The positive correlation between MEsalmon (module 7) and severe asthma was found to be strongest, while the negative correlation between MEsalmon and the control, as well as moderate asthma, was the strongest. Interestingly, the correlation between the two modules and phenotypes gradually changed. Through the STEM software, 24 genes were identified with gradually changing expression from healthy to severe asthma (Table S1). Surprisingly, the hub gene CEACAM6 of module 7 was also found in the STEM analysis results, where its expression gradually increased (Fig. 2C). Therefore, the genes in module 7 may directly affect the development of asthma, especially CEACAM6.

### Biological function and signal pathway of module genes

**Fig. 3:**
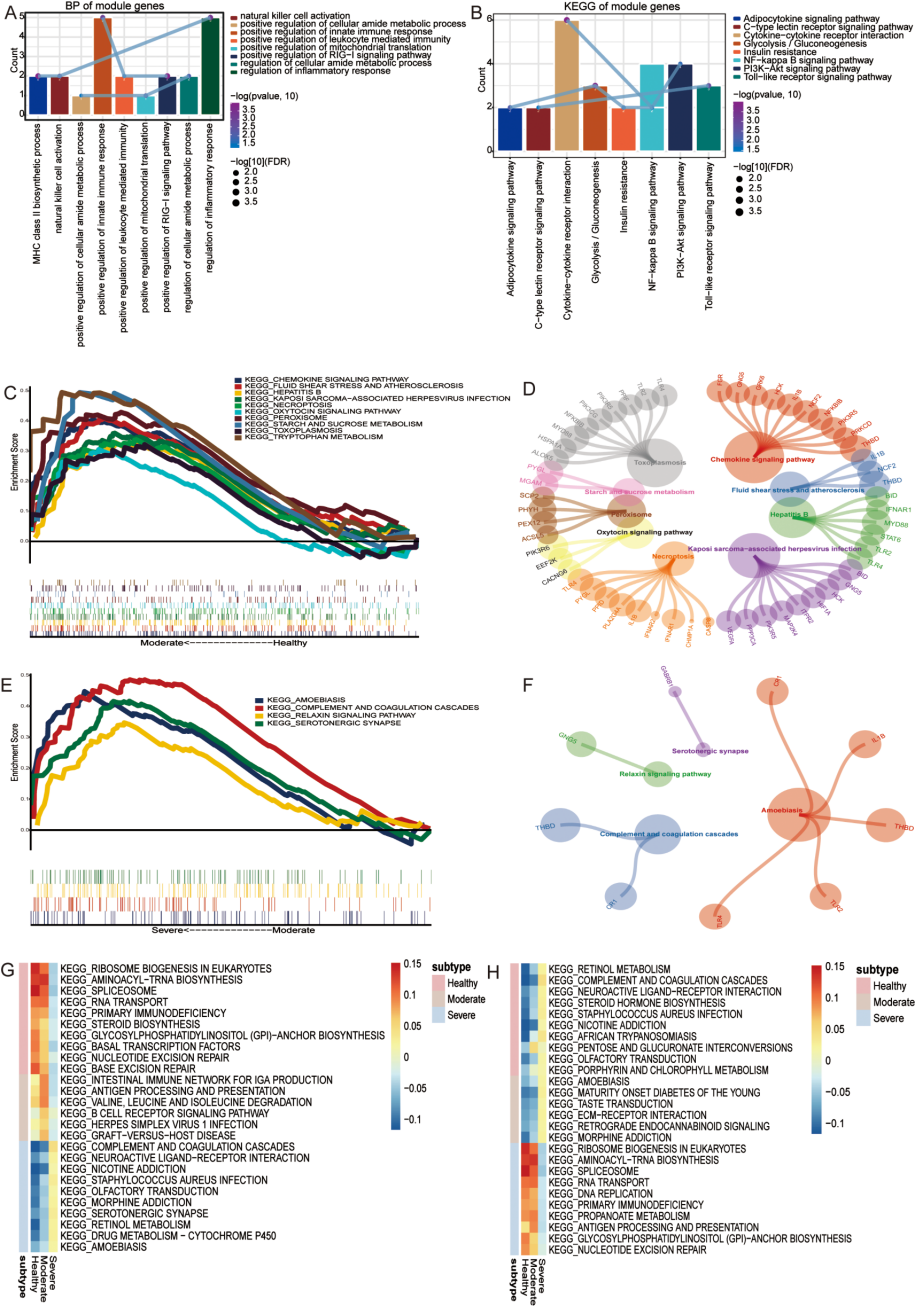
The biological function and signal pathway of module genes. (**A**) Biological processes in which module genes participate. (**B**) KEGG pathway in which module genes participate. The KEGG of GSEA in moderate asthma is the same as those of the enrichment (**C**) and genes involved in the same signaling pathway (**D**). The KEGG of GSEA in severe asthma is equivalent to those of the enrichment (**E**) and genes involved in the same signaling pathway (**F**). (**G**) The upregulated signaling pathway in healthy to moderate and to severe asthma. (**H**) The downregulated signaling pathway in healthy to moderate and to severe asthma

Furthermore, the enrichment of GO and KEGG of the module genes were analyzed, in which the module genes were found to be significantly enriched in 5599 biological processes (BP), 744 cell components (CC) and 1137 molecular functions (MF). In BP, these genes were mainly enriched in immune and inflammatory response (Fig. 3A). In addition, the module genes were found to be involved in 210 KEGG pathways. The main signal pathways enriched by the module genes were related with oxidative stress, including Toll-like receptor signaling pathway, Cytokine-cytokine receptor interaction, and NF-kappa B signaling pathway. (Fig. 3B). It was worth noting that according to the GSEA results, 10 signaling pathways related to moderate asthma were equivalent to the enrichment results (Fig. 3C) and involved multiple module genes (Fig. 3D). Moreover, four signal pathways related to severe asthma were equivalent to the enrichment results (Fig. 3E and F). These signaling pathways may play an important role in moderate and severe asthma, respectively. The results of subGSEA showed that the complement and coagulation cascades increased gradually in the course of asthma exacerbation, while ribosome biogenesis in eukaryotes and spliceosome decreased gradually (Fig. 3G and H).

### Verification of key pathway genes in public databases

**Fig. 4:**
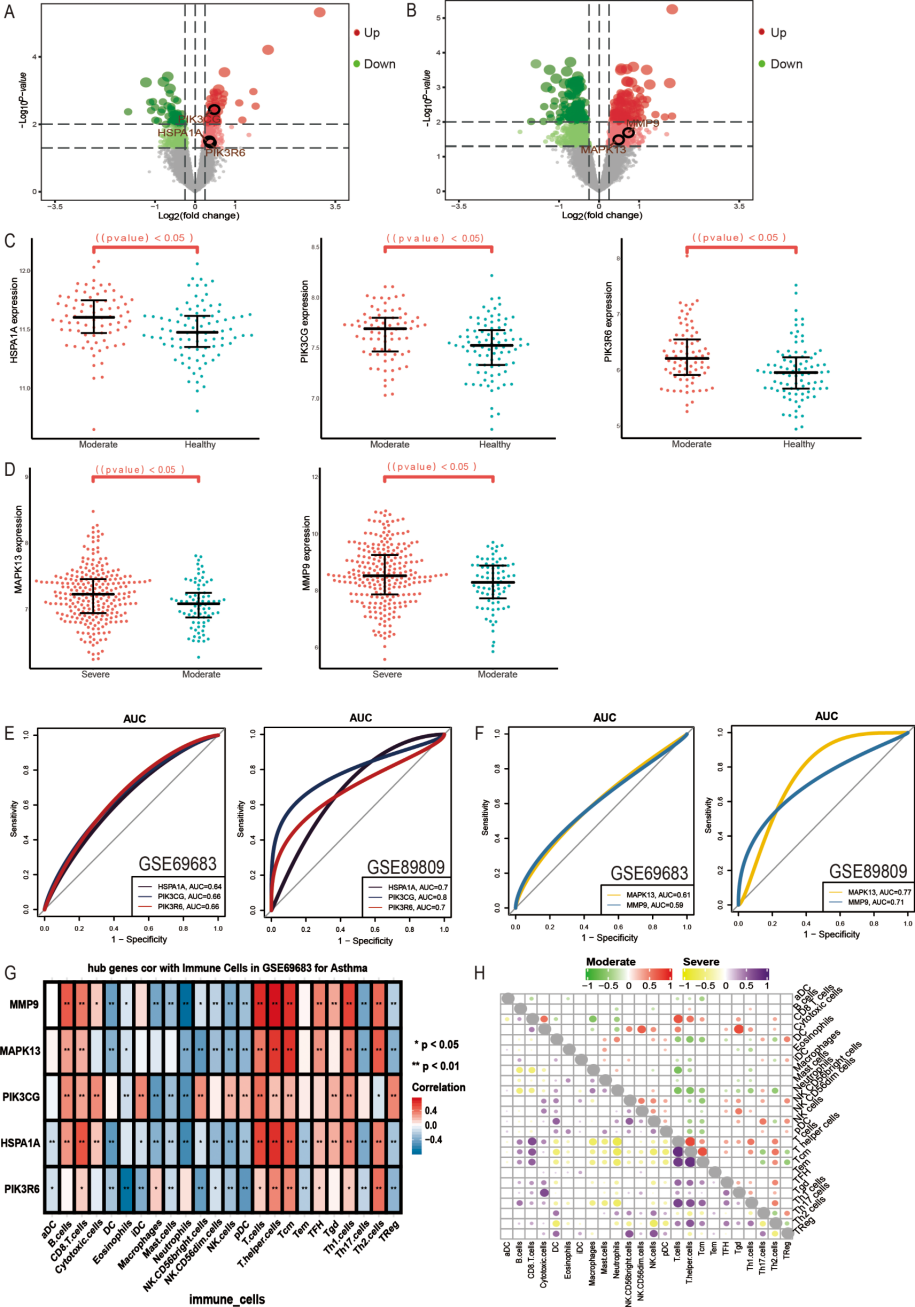
Verification of key genes. (**A**) DEGs between the moderate and control groups of epicellular cells in GSE89809. (**B**) DEGs between the severe and moderate groups of epicellular cells in GSE89809. (**C**) Expression levels of HSPA1A, PIK3CG and PIK3R6 in GSE89809. (**D**). Expression levels of MAPK13 and MMP9 in GSE89809. (**E**) AUC values of HSPA1A, PIK3CG and PIK3R6 in moderate of GSE69683 and GSE89809. (**F**) AUC values of MAPK13 and MMP9 in severe of GSE69683 and GSE89809. (**G**) Correlation between key genes and immune cells. (**H**) Correlation between immune cells in patients with severe and moderate asthma

To verify the above results, DEGs between the severe cell, the moderate cell and the control cell were analyzed in GSE89809 (Fig. 4A and B). The expression of HSPA1A, PIK3CG and PIK3R6 in the same 10 and 4 pathways was then verified (Fig. 4C), while the expression of MAPK13 and MMP9 related to severe asthma was also confirmed (Fig. 4D). The corresponding ROC curve showed that all five genes possessed a good AUC value, which may have clinical diagnostic ability (Fig. 4E and F). The enrichment analysis showed that the genes related to moderate and severe asthma were mainly related to immunity, therefore, the correlation between the five hub genes and immune cells were calculated (Fig. 4G). These genes were found to have the highest positive correlation with T helper cells, T cells and TCM. In severe asthma, T helper cells had a high positive correlation with TCM, while in moderate asthma, T helper cells had a high positive correlation with T cells (Fig. 4H). In addition, we also predict drugs regulated five genes using DGIdb database, and found 322 drugs targeted them (Table S2).

## Discussion

The pathophysiological mechanism of asthma is very complex, hence, WGCNA may serve as an effective tool for mining valuable data and analyzing complex genetic networks. In this study, WGCNA was used to construct a gene co-expression network to understand the progression of asthma, identifying changes in signal pathways in moderate to severe asthma through enrichment analysis. Key genes were verified using publicly available data.

Regarding the two stages of asthma, the expression of module genes changed significantly. The correlation between most modules and clinical phenotypes was gradually changing, indicating that the change in module genes was related to the development of asthma, especially module 3 and module 7. This was confirmed by the STEM results, where the hub gene CEACAM6 of module 7 was observed to be continuously upregulated in asthma. Various studies have confirmed the expression of CEACAM 6 (carcinoembryonic antigen related cell adhesion molecule 6) in the normal adult lung, participating in the innate defense of cells as well as the control of cell proliferation [15]. CEACAM6 transcription was found in the bronchial biopsy of asthmatic patients and was related to neutrophils [16].

The enrichment analysis showed that the genes of module 3 and module 7 were related to immunity and inflammatory response. The main role of T cells in asthmatic airways was found to be controlling the distribution of inflammatory cells [17]. The majority of mast cells in moderate allergic asthma were Th2 cell-dependent tryptase expression type [18]. In addition, module 3 genes were mainly involved in the negative regulation of TGF - β activation, while module 7 genes were mainly involved in the positive regulation of interleukin-1 (IL-1) and MHC class II. Transforming growth factor-β (TGF - β) is a pleiotropic cytokine involved in both suppressive and inflammatory response [19]. TGF - β was found to be the main mediator in promoting an inflammatory response as well as the remodeling of fibrous tissue in asthmatic lungs, however, its role as a therapeutic target remains controversial [20]. TGF - β could regulate Treg, Th17, NKT and CD8 + T cells and inhibit Th1 and Th2 differentiation [21]. Members of the IL-1 family were found to be closely related to damage of inflammation [22]. An increasing number of studies have shown that pro-inflammatory cytokines in the IL-1 family, especially IL-1α and IL-1β, were involved in the development of asthma [23]. In the lung tissues of asthmatic patients, the expression of MHC class II in airway epithelial cells was observed to be increased [24]. In particular, the present findings show that the expression of complex and coalescence cascades were not only present in the enrichment and GSEA results, but were also continuously upregulated in the development of asthma. The complement system is an important driver of inflammation, and excessive complement activation may lead to many inflammatory diseases, including asthma [25, 26].

Among the genes involved in these important signaling pathways, HSPA1A, PIK3CG and PIK3R6 seemed to be associated with moderate asthma, while MAPK13 and MMP9 appeared to be associated with severe asthma. The increase in HSPA1A levels was found to be related to the severity of asthma [27]. MAPK 13 exhibited more tissue-specific expression patterns and became a disease-specific p38MAPK drug target [28]. A study regarding a disease model among MAPK 13 knockout mice highlighted that the MAPK 13 dependent signaling pathway may lead to asthma and other diseases [29]. Persistent MMP-9 signaling was associated with tissue remodeling in asthmatic patients [30]. In severe asthma, which was dominated by neutrophils, the process of MMP-9 was found to be upregulated [31].

MMP-9 is also related to disease activity and severity in other inflammatory diseases [32]. For example, higher levels of MMP-9 have been found in patients with chronic spontaneous urticaria [33], atopic dermatitis [34], and allergic rhinitis [35]. This suggests that MMP-9 may play a broader role in inflammatory responses across various diseases, indicating its potential as a universal biomarker for inflammation severity and a target for therapeutic interventions.

PIK3CG and PIK3R6, though having no known role in asthma, was verified in the present analysis as well as other published datasets, which were used as potential novel biomarkers or therapeutic targets.

However, this study had certain limitations, such as only using ROC curves to predict the prognostic value of key genes. Additionally, a lack of experimental validation data, may have affected the obtained results. The prognostic role of these biomarkers in asthmatic patients should be further studied in order to explore the potential mechanisms and related pathways of these genes.

## Conclusion

Overall, this study discovered a total of five genes, including HSPA1A, PIK3CG, PIK3R6, MAPK 13 and MMP9, as potential biomarkers of moderate or severe asthma. CEACAM6 may serve as a therapeutic target in inhibiting the development of asthma. This study may provide a basis for the diagnosis and treatment of asthma in the future.

## Electronic supplementary material

Below is the link to the electronic supplementary material.

Supplementary Material 1

## Data Availability

The datasets generated during and/or analysed during the current study are available from the corresponding author on reasonable request.

## Abbreviations

JAK-STAT: Janus kinase/signal transducer and transcription
DEGs: Differentially expressed genes
GEO: Gene expression omnibus
WGCNA: Weighted gene co-expression network analysis
GS: Gene significance
MM: Module membership
GSEA: Gene set enrichment analysis
GO: Gene Ontology
KEGG: Kyoto Encyclopedia of Genes and Genomes
ROC: Receiver operating characteristic
BP: Biological processes
CC: Cell components
MF: Molecular functions
IL-1: Interleukin-1
TGF –β: Transforming growth factor-β
