# Supplementary material for: Identification of candidate genes and molecular mechanisms related to asthma progression using bioinformatics
Source: Sleep Breath. 2024 Aug 1;28(5):2237–46. doi: 10.1007/s11325-024-03122-0 (PMC11450000; doi:10.1007/s11325-024-03122-0)
Supplement: Supplementary file 1 — Supplementary Material 1 [file 11325_2024_3122_MOESM1_ESM.docx]

**Supplementary material**

**Figure S1.** Heatmap of differentially expressed genes in modules.

**Table S1.** STEM analysis revealed genes with a gradual change in expression from healthy to severe asthma.

**Table S2.** Drugs targeted to hub genes.
